# Generally recognized as safe (GRAS) *Lactococcus lactis* strains associated with *Lippia sidoides* Cham. are able to solubilize/mineralize phosphate

**DOI:** 10.1186/s40064-016-2596-4

**Published:** 2016-06-22

**Authors:** Jackeline Rossetti Mateus de Lacerda, Thais Freitas da Silva, Renata Estebanez Vollú, Joana Montezano Marques, Lucy Seldin

**Affiliations:** Instituto de Microbiologia Paulo de Góes, Universidade Federal do Rio de Janeiro, Rio de Janeiro, RJ CEP 21941-590 Brazil; Instituto de Ciências Biológicas, Universidade Federal do Pará, Belém, PA CEP 66075-900 Brazil; Laboratório de Genética Microbiana, Centro de Ciências da Saúde, Departamento de Microbiologia Geral, Instituto de Microbiologia Paulo de Góes, Universidade Federal do Rio de Janeiro, Bloco I, Ilha do Fundão, Rio de Janeiro, RJ CEP 21941.590 Brazil

**Keywords:** Phosphate solubilization, Phosphate mineralization, Generally recognized as safe (GRAS), *Lactococcus lactis*, Endophytes, *Lippia sidoides* Cham.

## Abstract

Eight strains isolated from the stems of *Lippia sidoides* were identified as belonging to *Lactococcus lactis*, a bacterial species considered as “generally recognized as safe”. Their capacity to solubilize/mineralize phosphate was tested in vitro with different inorganic and organic phosphorus (P) sources. All strains were able to solubilize calcium phosphate as an inorganic P source, and the best result was observed with strain 003.41 which solubilized 31 % of this P source. Rock phosphate, a mined rock containing high amounts of phosphate bearing minerals, was solubilized by five strains. When calcium phytate was the organic P source used, the majority of the strains tested showed phosphate mineralization activity. Moreover, all strains were able to solubilize/mineralize phosphate from poultry litter, a complex P source containing inorganic and predominantly organic P. The presence of genes coding for phytase and alkaline phosphatase was searched within the strains studied. However, only gene sequences related to alkaline phosphatase (*phoA* and *phoD*) could be detected in the majority of the strains (excepting strain 006.29) with identities varying from 67 to 88 %. These results demonstrate for the first time the potential of *L. lactis* strains for phosphate solubilization/mineralization activity using a broad spectrum of P sources; therefore, they are of great importance for the future development of more safe bioinoculants with possible beneficial effects for agriculture.

## Background

The species *Lactococcus lactis* belongs to the *Firmicutes* phylum and its strains are characterized by the production of lactic acid as the main end product of carbohydrate metabolism. *L. lactis* is one of the most important microorganisms in the dairy industry (Ward et al. 2002), and has “generally recognized as safe” (GRAS) status (Wessels et al. 2004). Although *L. lactis* is found in a wide range of environments and even in a great variety of traditional food products (Salama et al. 1995; Velly et al. 2014), this species has been recurrently found in different parts of various plants. They have already been isolated from the interior of aquatic plants (Chen et al. 2012), from stems of *Eucalyptus* (Procópio et al. 2009), from baby corn and fresh green peas (Alemayehu et al. 2014) and from the inner tissues and leaves of sugarcane (Beneduzi et al. 2013; Cock and de Stouvenel 2006, respectively). Likewise, da Silva et al. (2013) isolated from the Brazilian essential oil producing plant *Lippia sidoides* Cham. (pepper-rosmarin) eight bacterial endophytes identified as *L. lactis.* Recently, Golomb and Marco (2015) showed that certain strains of *L. lactis* are well adapted for growth on plants.

Strains growing in association with plants may exert positive effects on plant growth directly or indirectly through one or more mechanisms. For example, these strains can fix nitrogen, synthesize phytohormones and vitamins, improve nutrient uptake, solubilize inorganic phosphate and mineralize organic phosphate, among other direct benefits to the plant. Indirectly, some bacterial strains can produce siderophores and/or synthesize antimicrobial compounds decreasing or even preventing the deleterious effects of pathogenic microorganisms (Dobbelaere et al. 2003). However, to the best of our knowledge, only very few studies are available considering strains of *L. lactis* as potential plant growth promoters. Somers et al. (2007) and Grönemeyer et al. (2012) described the isolation of *L. lactis* strains showing significant plant growth promoting activity in greenhouse trials with cabbage and from agricultural crops in the Kavango region of Namibia, respectively. The ability to solubilize tricalcium phosphate has been demonstrated in *L. lactis* by growing the strains MH5-2 and MH5-5, isolated from mahangu, on bromophenol blue-containing Pikovskaya agar (Grönemeyer et al. 2012). However, their ability to solubilize/mineralize phosphate converting different sources of insoluble phosphates into available forms for plant has not been explored so far.

Phosphorus (P) is considered the second most important nutrient for plant nutrition. It is implicated in different plant processes and as an integral component of several plant structures such as phospholipids, proteins and nucleic acids (Balemi and Negisho 2012). Although P is abundant in soil, this nutrient is usually unavailable for plant uptake because it readily forms insoluble complexes with aluminium, iron, calcium and/or magnesium in soil (Vance et al. 2003; Altier et al. 2013). It is estimated that crop productivity is limited by P deficiency on more than 40 % of the world arable lands (Vance 2001). To maintain the crop yield, huge amounts of P containing fertilizers are usually applied worldwide. Moreover, global P reserves are being depleted at a higher rate demanding high energy costs and resulting in deleterious environmental impacts, such as eutrophication and carbon emissions (Balemi and Negisho 2012; Sharma et al. 2013).

Phosphate solubilizing/mineralizing bacteria are an environmental friendly alternative to the use of chemical P fertilizers (Richardson and Simpson 2011). Moreover, bacterial groups with GRAS status are of great interest as potential bioinoculants with low chances of causing plant or human diseases (Rosenblueth and Martínez-Romero 2006; Mendes et al. 2013). Therefore, the main purpose of this study was to test the *L. lactis* strains previously isolated from *L. sidoides* for their capacity of phosphate solubilization/mineralization of several inorganic and organic phosphate sources, such as calcium phosphate, aluminium phosphate, rock phosphate, phytate and poultry litter. The results obtained here meet the increased interest in the harnessing of microorganisms to support P cycling in agroecosystems.

## Methods

### Bacterial strains from *Lippia sidoides* Cham.

The bacterial strains (003.41, 006.27, 006.29, 006.30, 006.31, 104.3, 104.6 and 104.7), used in this study, have been previously isolated from the stems of three genotypes of *L. sidoides* (LSID003, LSID006 and LSID104) as described by da Silva et al. (2013). The different isolates were maintained both in BD™ Tryptic Soy Broth (TSB) agar-containing slants at room temperature and in TSB with 20 % glycerol at −80 °C.

### DNA extraction, PCR amplification, sequencing and phylogenetic analysis based on 16S rRNA

Genomic DNA was extracted from all bacterial strains using the protocol presented in da Silva et al. (2013). A partial sequence of the 16S rRNA gene (~1400 bp) was obtained by PCR amplification using the pair of universal primers pA (5′-AGAGTTTGATCCTGGCTCAG-3′) and pH (5′-AAGGAGGTGATCCAGCCGCA-3′) and the conditions described in Massol-Deya et al. (1995). Negative controls (without DNA) were run in all amplifications. The PCR products were sequenced using Macrogen (South Korea) facilities, and the partial 16S rRNA gene sequences were identified using the BLAST-N tool (www.ncbi.nlm.nih.gov/blast) on the National Center for Biotechnology Information (NCBI) website using the GenBank non-redundant database. The resulting sequences were aligned to sequences from *Lactococcus* type strains available in the GenBank database using the CLUSTALW tool (Thompson et al. 1994). MEGA 6 software (Tamura et al. 2013) was used to construct a phylogenetic tree based on the 16S rRNA gene sequences using the neighbor-joining method and Jukes–Cantor distances. Bootstrap analyses were performed with 1000 repetitions, and only values higher than 50 % are shown in the phylogenetic tree. The sequences generated in this study were deposited in NCBI GenBank under accession numbers KU639597- KU639604.

### PCR amplification of alkaline phosphatase and phytase encoding genes

Fragments of alkaline phosphatase and phytase genes were amplified by PCR using the pair of primers ALPS-R1101 (5′-GAGGCCGATCGGCATGTCG-3′) and ALPS-F730 (5′-CAGTGGGACGACCACGAGGT-3′; Sakurai et al. 2008) and BPP-F (5′-GACGCAGCCGAYGAYCCNGCNITNTGG-3′) and BPP-R (5′-CAGGSCGCANRTCIACRTTRTT-3′; Huang et al. 2009), respectively.

The products obtained were visualized by 1.4 % agarose gel electrophoresis in TBE buffer (Sambrook et al. 1989) at 80 V for 3–4 h at room temperature, and stained with ethidium bromide. The PCR products were then sequenced using Macrogen facilities, and the sequences were further compared to those previously deposited at the GenBank database using the BLAST-N tool.

### Phosphate solubilization/mineralization tests in liquid medium

The ability of the isolates to solubilize inorganic and mineralize organic phosphates in liquid medium was tested as described by Nautiyal (1999). Four different P sources (calcium phosphate, aluminium phosphate, rock phosphate and poultry litter) were used. The chemical composition of the poultry litter was (per g kg^−1^): Ca^2+^—0.39, Mg^2+^—0.43, K^+^—21.1, P—7.0, Al^3+^—0.65, Na^+^—3.5, and 0.86 % of organic carbon. Bacterial cultures were grown in the National Botanical Research Institute’s phosphate growth medium (NBRIP—containing in 50 ml: glucose, 0.5 g; MgCl_2_·6H_2_O, 0.25 g; MgSO_4_·7H_2_O, 0.0125 g; KCl, 0.01 g and (NH_4_)_2_SO_4_, 0.005 g) with the addition of 0.25 g of each P source. The cultures were incubated at 28 °C for 10 days with shaking (180 rpm). After incubation, the pH was measured using a pH Meter (PHI510, Beckman Coulter, California, USA) and the concentration of soluble P was determined as described by Murphy and Riley (1962).

### Phosphate mineralization tests in solid medium

The ability of the isolates to mineralize organic phosphate was tested as described by Rosado et al. (1998). Calcium phytate was used as the P source in the culture medium (glucose 1 %, (NH_4_)_2_SO_4_ 0.05 %, KCl 0.02 %, MgSO_4_·7H_2_O 0.01 %, calcium phytate 0.2 %, yeast extract 0.05 %, agar 1.5 % (w/v), MnSO_4_ 5 mg l^−1^, FeSO_4_ 5 mg l^−1^). The presence of clear zones around the bacterial colonies after incubation for 10 days at 32 °C was indicative of positive organic phosphate mineralization. The ratio between the diameters of each halo and the corresponding colony was determined as described by Hankin and Anagnostakis (1975).

### Statistical analysis

The phosphate mineralization/solubilization within the *L. lactis* strains was compared with one-way ANOVA followed by Tukey’s pairwise test. In case of unequal variances Welch F test was used, instead of ANOVA. All statistical analyses were computed using PAST software (Hammer et al. 2001).

## Results

**Fig. 1:**
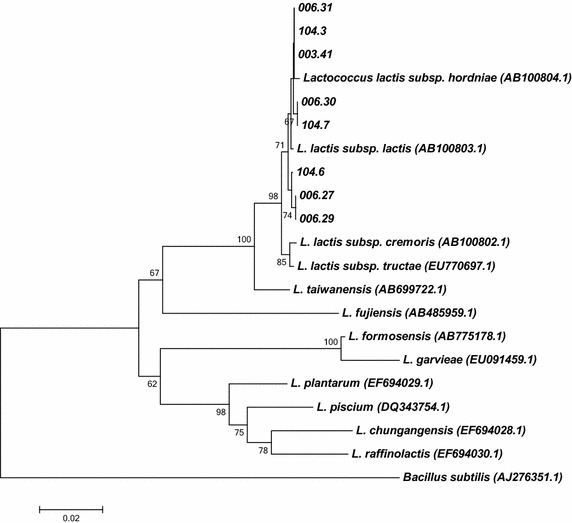
Phylogenetic tree based on the *rrs* gene sequence (~1400 bp) showing the relationship between the *Lactococcus* strains studied here and the other members of the genus. The tree was constructed based on Jukes–Cantor distance using the neighbor joining method. Bootstrap analyses were performed with 1000 repetitions and only values higher than 50 % are shown. The Genbank accession numbers of the *Lactococcus* species are shown in parentheses

Eight different bacterial strains (as determined by BOX-PCR) previously isolated from *L. sidoides* stems and molecularly identified as *L. lactis* (*rrs* gene fragment of about 800 bp; da Silva et al. 2013) were selected among the other isolates based on their potential for being GRAS. In this study, a phylogenetic tree based on their *rrs* gene sequences (approximately 1400 bp) was constructed and their previous identification was confirmed (Fig. 1).

**Fig. 2:**
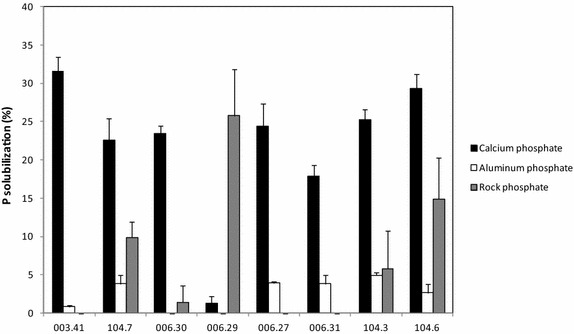
Percentages of solubilized phosphate using different P sources (calcium phosphate, aluminium phosphate and rock phosphate)

To test the P solubilization capacity of the eight selected strains, two chemically defined sources—calcium phosphate and aluminium phosphate—were first used (Fig. 2). All strains were able to solubilize calcium phosphate, varying from less than 2 to 31 % of this P source (strains 006.29 and 003.41, respectively). The other six strains tested showed intermediate results (18–29 %). The difference observed among the solubilization percentages obtained for strains 104.3, 006.31, 006.27, 006.30 and 104.7 were not statistically significant based on Tukey’s pairwise test (p < 0.05). Oppositely, either low solubilization percentages or no solubilization capacity (strains 006.29 and 006.30) were observed when aluminium phosphate was used as an inorganic P source (Fig. 2). The best result was observed with strain 104.3 which solubilized approximately 5 % of the phosphate available (Fig. 2).

Rock phosphate, a mined rock that contains high amounts of phosphate bearing minerals, was also used to test the ability of the eight strains to solubilize P. In general, the strains tested were more efficient in solubilizing rock phosphate than aluminium phosphate, but less efficient when compared to calcium phosphate solubilization (Fig. 2). Surprisingly, the highest percentage (approximately 26 %) of rock P solubilized was achieved by the strain 006.29, which was unable to solubilize aluminium phosphate and presented a low solubilization percentage when calcium phosphate was used as a P source. Oppositely, the strains 003.41, 006.27 and 006.31 were unable to solubilize rock phosphate (Fig. 2).

**Fig. 3:**
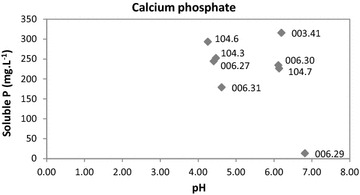
Correlation between the percentage of phosphate solubilized and the pH values of the media after 10 days of culture incubation. Calcium phosphate was used as the P source

One of the most important mechanisms responsible for inorganic phosphate solubilization is the production of organic acids by bacterial P solubilizers. To detect the presence of acids during calcium phosphate solubilization, pH was measured after 10 days of culture incubation (just before the P solubilization measurements). The initial pH of the medium varied from 6.5 to 7.0. No straight correlation could be observed between acid production and calcium phosphate solubilization. For example, strains 003.41 and 006.29 showed the highest (315.6 mg l^−1^) and the lowest (13.3 mg l^−1^) phosphate solubilization values, respectively, but the pH values were almost the same as in the beginning of the culturing procedure in both strains (Fig. 3). A decrease in the pH values was observed for the remaining strains tested and the calcium phosphate solubilization values varied among them (Fig. 3).

**Fig. 4:**
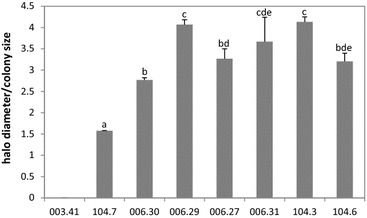
Phosphate mineralization activity of the different *L. lactis* strains determined by the semi-quantitative method described in the materials and methods. Calcium phytate was used as the P source. *Different letters* indicate significant differences of means in pairwise comparisons (Tukey test; p < 0.05)

Phosphate mineralization is another mechanism responsible for P availability in soil besides phosphate solubilization. Therefore, a semi-quantitative method using an organic chemical defined P source (calcium phytate) was used to test the phosphate mineralization capacity of the eight *L. lactis* strains. The majority of the strains tested (7 strains) showed phosphate mineralization activity, excepting strain 003.41. Some phosphate mineralizing strains showed differences in their phosphate mineralization capacity, which were statistically significant based on Tukey’s pairwise test (p < 0.05) (Fig. 4).

**Fig. 5:**
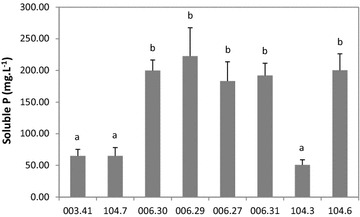
Phosphate mineralization/solubilization activity of the different *L. lactis* strains tested as described by Nautiyal (1999). Poultry litter was used as the P source. *Different letters* indicate significant differences of means in pairwise comparisons (Tukey test; p < 0.05)

Poultry litter, a complex P source containing inorganic and organic phosphates, was also included here in order to test the mineralization/solubilization activity of the eight selected strains. The highest activities were obtained with strains 006.30, 006.29, 006.27, 006.31 and 104.6 (Fig. 5). Based on Tukey’s pairwise test, no significant differences were found among these five strains and also among the remaining three strains—003.41, 104.7 and 104.3 (Fig. 5).

The mineralization activity can be associated with the presence of enzymes such as phytase and/or alkaline phosphatase. The presence of genes coding for these proteins were searched within the genomes of the studied strains. When specific primers were used for phytase coding genes, no PCR products were observed. On the other hand, gene sequences related to alkaline phosphatase (*phoA* and *phoD*) could be detected in the majority of the strains (excepting strain 006.29) with identities varying from 67 to 88 %.

## Discussion

Different endophytic bacteria are reported to increase the solubility of phosphorus and thus promote the growth of the host plants (Ryan et al. 2008). In this study, it was demonstrated for the first time that strains of *Lactococcus lactis*, a well-established group of GRAS bacteria, previously isolated from *L. sidoides* inner tissues were capable of mineralizing and/or solubilizing different P sources in vitro.

Strains belonging to the genera *Pseudomonas* and *Bacillus* are considered the most frequently found bacteria showing the capability of mineralizing and/or solubilizing phosphate in association with plants (Sharma et al. 2013). However, other genera such as *Rhizobium* (Sridevi et al. 2007), *Azotobacter* (Kumar et al. 2001), *Enterobacter*, *Pantoea*, *Klebsiella* (Chung et al. 2005), among other bacteria have also been reported as phosphate solubilizers/mineralizers. The few studies demonstrating the presence of *Lactococcus* able to mineralize and/or solubilize phosphate using a cultivation-dependent approach could be explained by either the low number of strains found inside the plants (or in the rhizosphere) or their slower growth compared to other bacterial genera. Inside the *L. sidoides* plants, Gammaproteobacteria appear to predominate which made it difficult to recover members of the bacterial community found in low numbers such as *L. lactis* (da Silva et al. 2013).

The eight *L. lactis* strains tested here were able to solubilize calcium phosphate and to solubilize/mineralize phosphate from poultry litter, a complex P source containing inorganic and predominantly organic P. Moreover, only one strain tested did not show any phosphate mineralization activity using calcium phytate as the organic P source. In fact, it has been previously demonstrated that other lactic acid bacteria isolated from sourdoughs exhibited a considerable phytate degrading capacity (De Angelis et al. 2003). The broad spectrum of phosphate utilization presented by these strains make them attractive as candidates for potential use in different types (alkaline, acidic or organic-rich) of soil, as suggested by Bashan et al. (2013). In addition, lactic acid produced by *Lactococcus* may have antimicrobial activity, exerting positive effects on plant growth indirectly. However, the isolates should be tested on a model plant as the ultimate test for potential phosphate solubilization (or mineralization) and/or for other plant growth promoting characteristics.

Inorganic phosphate solubilization by bacteria occurs mainly by the production of low molecular weight organic acids (Kpomblekou and Tabatabai 1994), and the lowering in pH of the medium suggests the release of these organic acids (Maliha et al. 2004). In this study, this was not the case for the strain 003.41 which showed the highest phosphate solubilization value (315.6 mg l^−1^) and a stable pH during the culturing procedure. Chelation of cations (Fe, Al and Ca) bound to phosphate, converting it into soluble forms may be other mechanism used for the release of phosphate (Sharma et al. 2013).

Organic phosphate mineralization by bacteria takes place due to the presence of particular enzymes. Plants are not able to acquire P directly from phytate (Richardson and Simpson 2011); therefore, phytase-producing microorganisms are the key drivers in regulating the mineralization of phytate in soil. In this study, none of the *L. lactis* strains tested seems to harbor phytase coding genes, as no PCR products were observed when specific primers were used. It is also possible that the primers used were not universal enough to target the phytase coding genes of the *L. lactis* strains studied here. Oppositely, gene sequences related to alkaline phosphatase (*phoA* and *phoD*) could be detected in seven of the strains tested. Alkaline phosphatases are more abundant in neutral and alkaline soils, and plants rarely produce large quantities of these enzymes (Sharma et al. 2013). Therefore, association of the *L. lactis* strains tested here with plants may be a potential niche for their use. Nevertheless, the presence of alkaline phosphatases coding genes only suggests that this can be one of the mechanisms of phosphate mineralization and further studies are necessary.

In conclusion, eight GRAS *L. lactis* strains are now available for future research to deeply understand their contribution to the cycling of P in soil–plant systems and for their eventual application under field conditions. The results presented here are of great importance as a first step for the development of more safe bioinoculants capable of mineralizing/solubilizing phosphate for the benefit of plants.
